# How Significant Are Differences Obtained by Neglecting Correlations When Testing for Deformation: A Real Case Study Using Bootstrapping with Terrestrial Laser Scanner Observations Approximated by B-Spline Surfaces

**DOI:** 10.3390/s19173640

**Published:** 2019-08-21

**Authors:** Gaël Kermarrec, Jens-André Paffenholz, Hamza Alkhatib

**Affiliations:** Geodetic Institute, Leibniz University Hannover, Nienburger Str. 1, 30167 Hannover, Germany

**Keywords:** 3D point clouds, B-spline surface approximation, terrestrial laser scanner, laser tracker, deformation analysis, bootstrap, test statistics, mathematical correlation, intensity model

## Abstract

B-spline surfaces possess attractive properties such as a high degree of continuity or the local support of their basis functions. One of the major applications of B-spline surfaces in engineering geodesy is the least-square (LS) fitting of surfaces from, e.g., 3D point clouds obtained from terrestrial laser scanners (TLS). Such mathematical approximations allow one to test rigorously with a given significance level the deformation magnitude between point clouds taken at different epochs. Indeed, statistical tests cannot be applied when point clouds are processed in commonly used software such as CloudCompare, which restrict the analysis of deformation to simple deformation maps based on distance computation. For a trustworthy test decision and a resulting risk management, the stochastic model of the underlying observations needs, however, to be optimally specified. Since B-spline surface approximations necessitate Cartesian coordinates of the TLS observations, the diagonal variance covariance matrix (VCM) of the raw TLS measurements has to be transformed by means of the error propagation law. Unfortunately, this procedure induces mathematical correlations, which can strongly affect the chosen test statistics to analyse deformation, if neglected. This may lead potentially to rejecting wrongly the null hypothesis of no-deformation, with risky and expensive consequences. In this contribution, we propose to investigate the impact of mathematical correlations on test statistics, using real TLS observations from a bridge under load. As besides TLS, a highly precise laser tracker (LT) was used, the significance of the difference of the test statistics when the stochastic model is misspecified can be assessed. However, the underlying test distribution is hardly tractable so that only an adapted bootstrapping allows the computation of trustworthy *p*-values. Consecutively, the extent to which heteroscedasticity and mathematical correlations can be neglected or simplified without impacting the test decision is shown in a rigorous way, paving the way for a simplification based on the intensity model.

## 1. Introduction

Most users of terrestrial laser scanner (TLS) observations analyse the recorded 3D point clouds in software such as CloudCompare (www.cloudcompare.org/), 3DReshaper (Hexagon Metrology, Cobham, Wimborne Minster, UK) or Geomagic Studio (3DSystems, Rock Hill, SC, USA). Such software allows to visualize maps of deformation, which are mainly based on the computation of a predetermined distance. This latter depends on the application under consideration and can be, e.g., Cloud to Cloud (C2C), Cloud to Mesh (C2M), Mesh to Mesh (M2M) or M3C2 for CloudCompare, see Holst and Kuhlmann [1] for a short comparison of the different strategies.

The main drawback of this approach is the impossibility to carry out a rigorous statistical test for deformation: no decision based on a statistical approach can be taken, whether a null hypothesis stating that no deformation magnitude occurs between two epochs can be rejected or not for a predefined significance level. Alternatively, approximations of the point clouds with mathematical models such as B-spline surfaces allow to derive such statistical tests. However, usual congruency tests (Pelzer [2]) are irrelevant in the specific case of B-spline surface approximation. Indeed, different numbers of parameters called control points (CP) may have to be estimated at the two different epochs to fit optimally the point clouds in least-squares (LS) sense. Consecutively, Zhao et al. [3] and Kermarrec et al. [4] proposed a specific procedure for testing deformation based on gridded B-spline surface approximations from TLS point clouds. The distribution of the apriori test statistics could be rigorously determined as corresponding to a chi-squared distribution.

Unfortunately, test statistics are known to be strongly influenced by the underlying stochastic model of the observation’s errors (see, e.g., Kermarrec et al. [5]). For both a trustworthy test decision and LS solution, an optimal description of the stochasticity is needed as any inaccuracies may affect further risk analysis. TLS raw observations (range, horizontal and vertical angles) are known to be heteroscedastic, i.e., depending on the scanning geometry (Soudarissane et al. [6]) or the properties of the scanned objects (Wujanz et al. [7]). Furthermore, correlations between range measurements are expected to affect the computed deformation magnitude (Holst et al. [1], Jurek et al. [8]). Assuming homoscedasticity is, thus, a strong assumption, which weaken the test statistics, when B-spline surfaces are estimated from scattered and noisy point clouds. Moreover, due to the transformation of the raw observations from polar to Cartesian coordinates to perform the LS approximation, mathematical correlations are introduced. Accounting for such specific correlations in the adjustment lead to fully populated variance covariance matrix (VCM), similarly to temporal correlations (Kermarrec et al. [9]). Such matrices are less easy to handle than their diagonal counterpart, particularly when their inverse is involved in the estimations. Neglecting them is, thus, a tempting alternative to avoid computational burden. Consecutively, an incorrect rejection of the null hypothesis may happen, when the test statistics corresponding to a simplified stochastic model are close to the predefined critical value of the test. Accounting for both heteroscedasticity and mathematical correlations is expected to avoid such challenging situations. However, the difference between the test statistics obtained with different stochastic models may not be significant enough to allow for such a strong conclusion. Thus, it may be totally sufficient to use diagonal VCM for a trustworthy test decision, which would simplify grandly the computation in case of large matrices.

In this contribution, we propose to investigate the significance of the difference between the test statistics obtained with different VCM, focusing on gradually neglecting mathematical correlations. Thanks to a case study with real observations for which reference values of deformation were obtained with a highly precise LT, we aim to point out the undertaken risk—or not—when mathematical correlations or heteroscedasticity are neglected.

The *p*-value provides information about the significance of the difference (see Wasserstein and Lazar [10] for a didactical explanation of the *p*-values and their limitations). Thus, by analysing them, it is possible to confirm and extend the empirical findings of simulations to a real case study within a rigorous framework. Using deformation magnitude from a LT as a reference, we will, in this contribution, show how neglecting mathematical correlations affect or not these *p*-values. Because the distribution of the test statistics is hardly tractable, we propose to use an innovative bootstrap approach (Kargoll et al. [11]).

The remainder of the paper is as follows: in a first part, we will shortly present how B-spline surfaces can be approximated from scattered data by means of a LS adjustment. Focusing on deformation analysis, a specific test procedure will be described. We will explain the concept of mathematical correlations and heteroscedasticity and present simplifications of the stochastic model. A bootstrap testing approach will be introduced to determine the *p*-values for different stochastic models. A third part is devoted to the application of the theoretical developments using TLS and LT observations from a bridge under load. We will conclude by analysing if and when mathematical correlations can be neglected.

## 2. Mathematical Background

In this section, we propose to explain the concept of the approximation of TLS point clouds with B-spline surfaces. The second part of this section describes the stochastic model of the TLS observations, with a focus on mathematical correlations. In a third part, we will develop our strategy to test for deformation of gridded surfaces. A novel bootstrap approach will be presented to assess the extent to which differences of the chosen test statistic obtained with simplified stochastic models can be considered as significant or not.

### 2.1. Approximation of TLS Point Cloud with B-Spline Surfaces

For the sake of shortness, we will focus only on the main steps involved in the approximation of point clouds with parametrized B-spline surfaces. Interested readers can find more specific information, e.g., in Bureick et al. [12].

The first step of an approximation with a B-spline surface starts with the parametrization of the point cloud. Two location parameters u and v have to be associated with each point, which will be used to construct a knot vector for the spline approximation. Although iterative parameter correction procedures have been suggested, the chord length method mentioned in Piegl and Tiller [13] gives satisfactory results for regularly and rectangular shaped point clouds. Dealing in this contribution with rectangular patches of homogeneous objects, we will make use of this strategy. Please note that for more complicated surfaces, the choice of the parameters may largely affect the results of the approximation (see, e.g., Ma and Kruth [14] and the references therein).

The parametric B-spline surface $S_{\left(u,v\right)}$ is expressed as:

(1) $$S_{\left(u,v\right)}=\sum_{i=0}^{n}\sum_{j=0}^{m}B_{i,p,t^{\left(u\right)}}\left(u\right)B_{j,q,t^{\left(v\right)}}\left(v\right)p_{ij}$$

where $B_{i,p}=B_{i,p,t^{\left(u\right)}}$ is the B-spline function of degree p in the direction of the surface parameter u depending on the non-decreasing sequence of real numbers $t^{\left(u\right)}={\left(t_{i}^{\left(u\right)}\right)}_{i=1}^{n+d+1}$, called knots. $B_{j,q}=B_{j,q,t^{\left(v\right)}}$ is the B-spline function of degree q in the direction of the surface parameter v depending on the non-decreasing sequence of real numbers $t^{\left(v\right)}={\left(t_{j}^{\left(v\right)}\right)}_{j=1}^{r+d+1}$. $B_{i,p},B_{j,q}$ are given by the recurrence relation (de Boor [15]), *i* is varied from 0 to n, the number of CP in the u-direction, whereas *j* is varied from 0 to m, the number of CP in the v-direction. In this contribution, we will take a degree of p=q=3 for the B-spline function, which corresponds to cubic B-splines known for their smoothness properties. We solve the determination of an optimal knot vector using the knot placement technique as described in Piegl and Tiller [13].

The parameter vector p of size (n+1)(m+1) contains the coordinates of the CP, which are weighting factors of the B-spline functions. They are defined in ${\mathbb{R}}^{3}$ by their Cartesian coordinates. The estimation of the coordinates of the CP is the central part of the approximation of scattered point clouds with B-spline surfaces and can be performed by minimizing the difference between the true and computed observations in a LS sense. In that case, the error term v=l−Ap is minimized by searching:

(2) $$\underset{p\in {\mathbb{R}}^{3}}{\min}{\left\|Ap-l\right\|}_{\Sigma }^{2}$$

We define the observation vector l of size $\left(n_{obs},3\right)$ as pointwise sorted and expressed in Cartesian coordinates. The deterministic design matrix A of size of $\left(3n_{obs},\left(n+1\right)\left(m+1\right)\right)$ is built based on the tensor product of the B-spline functions. We let $E\left(v\right)=0,\;E\left(vv^{T}\right)=\Sigma_{0}=\sigma_{0}^{2}Q_{0}$, $\Sigma_{0}$ is the true VCM of the error term, $\sigma_{0}^{2}$ the apriori variance factor and $Q_{0}$ the true Variance Cofactor Matrix of the error term. E(•) is the expectation operator. Interested readers should refer to Zhao et al. [3] for the detailed setting of the matrix.

The coordinates of the estimated CP are given by the generalized LS estimator from ${\hat{p}}_{0}={\left(A^{T}\Sigma_{0}^{-1}A\right)}^{-1}A^{T}\Sigma_{0}^{-1}l$ of size (3(n+1)(m+1),1). The aposteriori variance factor is given by ${\hat{\sigma }}_{0}^{2}=\frac{{\hat{v}}_{0}{}^{T}\Sigma_{0}^{-1}{\hat{v}}_{0}}{n_{obs}-3\left(n+1\right)\left(m+1\right)}$ with ${\hat{v}}_{0}=A{\hat{p}}_{0}-l$ being the residuals of the adjustment. Since the true $\Sigma_{0}$ is unknown, it is replaced by its estimate $\hat{\Sigma }$. The corresponding estimator reads thus:

(3) $$\hat{p}={\left(A^{T}{\hat{\Sigma }}^{-1}A\right)}^{-1}A^{T}{\hat{\Sigma }}^{-1}l$$

and the aposteriori variance factor is:

(4) $${\hat{\sigma }}^{2}=\frac{{\hat{v}}^{T}{\hat{\Sigma }}^{-1}\hat{v}}{n_{obs}-3\left(n+1\right)\left(m+1\right)}$$

with $\hat{v}=A\hat{p}-l$.

The apriori VCM of the estimates is given by:

(5) $${\hat{\Sigma }}_{\hat{p}\hat{p}}={\left(A^{T}{\hat{\Sigma }}^{-1}A\right)}^{-1}$$

and the corresponding cofactor matrix by $Q_{\hat{p}}{}_{\hat{p}}={\left(A^{T}{\hat{P}}^{-1}A\right)}^{-1}$ with $\hat{\Sigma }=\sigma_{0}^{2}\hat{Q}$.

The aposteriori VCM of the estimates reads:

(6) $${\hat{\Sigma }}_{\hat{p}\hat{p},post}={\hat{\sigma }}^{2}{\left(A^{T}{\hat{Q}}^{-1}A\right)}^{-1}$$

### 2.2. Determination of the Optimal Number of CP by Information Criteria

The number of CP to estimate can be iteratively adjusted by using information criteria (see, e.g., Alkhatib et al. [16] for the specific application of information criteria to B-spline surface approximations and the references therein):

(i) the Akaike information criterion (AIC), which minimizes the Kullback-Leibler divergence of the assumed model from the data-generating model, or
(ii) the Bayesian information criterion (BIC), which assumes that the true model exists and is thus more adequate for large samples.
  They are defined as:
  (7) $$\begin{array}{l} AIC=-2\left[l\left(\hat{p}\right)\right]+2n_{obs}\\ BIC=-2\left[l\left(\hat{p}\right)\right]+\log\left(3\left(n+1\right)\left(m+1\right)\right)n_{obs} \end{array}$$
  where we call $l\left(\hat{p}\right)$ the log-likelihood of the estimated parameters. Using this formulation, a minimum is searched, which corresponds to the optimal number of CP to estimate.

### 2.3. Stochastic Model for TLS Observations

Unfortunately, the Cartesian coordinates of the point cloud are not directly measured by a TLS. Thus, setting up $\hat{\Sigma }$ involves some knowledge about the stochastic properties of the original raw TLS observations, which are made of the range r expressed in (m), as well as horizontal and vertical angles, which we will call HA and VA respectively, expressed in (°).

These measurements are heteroscedastic, i.e., they have different variances $\sigma_{r}^{2},\sigma_{HA}^{2},\sigma_{VA}^{2}$ for each point of the point cloud. The angle variances are often assumed to be constant and their values are based on manufacturer datasheets (Boehler and Marbs [17]). On the contrary, $\sigma_{r}$ is expected to have a stronger point dependency. Influencing factors are exemplarily the range itself. In addition, properties of the reflected object and eventually atmospheric transmission (Soudarissanane et al. [6], Zamecnikova et al. [18]) can influence $\sigma_{r}^{2}$.

These dependencies are summarized in the signal to noise ratio (Hebert and Krotkov [19]). Thus, an empirical proposal based on the intensity values of the backscattered signal has been proposed to model the range variance (Wujanz et al. [16,20]). Justified by the small and homogeneous surfaces under consideration in this contribution, we will use the simplification of Kermarrec et al. [21]. Thus, we replace the point-wise standard deviation by a global value, which is computed using the mean of the intensity values of the object, i.e.,:

(8) $$\sigma_{r,mean}=\beta {\left(\bar{I}nt\right)}^{\alpha }$$

where $\bar{I}nt$ is the mean of the intensities of the reflected object expressed in Increment [Inc]. The three parameters α, β and c of the power law function can be determined empirically in controlled experiment by regression analysis for different laser scanners. For the TLS Zoller+Fröhlich (Z+F) Imager 5006 under consideration in this contribution, we will assume a root mean square of 7° for both angles as well as [α,β,c]=[−0.57,1.6,0].

Consecutively, the diagonal VCM ${\hat{\Sigma }}_{i,ori}$ for point *i* of the raw TLS measurements can be built based on these estimated variances and reads:

$${\hat{\Sigma }}_{i,ori}=\left[\begin{array}{ccc} \sigma_{i,r}^{2} & 0 & 0\\ 0 & \sigma_{i,HA}^{2} & 0\\ 0 & 0 & \sigma_{i,VA}^{2} \end{array}\right]$$

The whole matrix is easily built as ${\hat{\Sigma }}_{ori}=\left[\begin{array}{ccc} {\hat{\Sigma }}_{1} & 0 & 0\\ 0 & \ldots & 0\\ 0 & 0 & {\hat{\Sigma }}_{n_{obs}} \end{array}\right]$.

### 2.4. Mathematical Correlations

#### 2.4.1. Fully Populated VCM

As aforementioned, a B-spline approximation involves the Cartesian coordinates of the point cloud. By means of the error propagation law, the corresponding VCM $\hat{\Sigma }$ is obtained by the error propagation law:

(9) $$\hat{\Sigma }=F{\hat{\Sigma }}_{ori}F^{T}$$

The matrix F contains the derivatives of the point coordinates with respect to the range and angles and reads for one point *i*:

$$F_{i}=\left[\begin{array}{ccc} \sin\left(VA_{i}\right)\cos\left(HA_{i}\right) & r_{i}\cos\left(VA_{i}\right)\cos\left(HA_{i}\right) & -r_{i}\sin\left(VA_{i}\right)\sin\left(HA_{i}\right)\\ \sin\left(VA_{i}\right)\sin\left(HA_{i}\right) & r_{i}\cos\left(VA_{i}\right)\sin\left(HA_{i}\right) & r_{i}\sin\left(VA_{i}\right)\cos\left(HA_{i}\right)\\ \cos\left(VA_{i}\right) & -r_{i}\sin\left(VA_{i}\right) & 0 \end{array}\right]$$

As a consequence, the corresponding variances for one point in Cartesian coordinates of the point clouds are:

$$\begin{array}{l} \sigma_{X}^{2}={\left(r\cos\left(HA\right)\sin\left(VA\right)\right)}^{2}\sigma_{HA}^{2}+{\left(r\sin\left(HA\right)\sin\left(VA\right)\right)}^{2}\sigma_{VA}^{2}+{\left(\cos\left(HA\right)\sin\left(VA\right)\right)}^{2}\sigma_{r}^{2}\\ \sigma_{Y}^{2}={\left(r\sin\left(HA\right)\cos\left(HA\right)\right)}^{2}\sigma_{HA}^{2}+{\left(r\cos\left(HA\right)\sin\left(VA\right)\right)}^{2}\sigma_{VA}^{2}+{\left(\sin\left(HA\right)\sin\left(HA\right)\right)}^{2}\sigma_{r}^{2}\\ \sigma_{z}^{2}={\left(r\sin\left(VA\right)\right)}^{2}\sigma_{HA}^{2}+{\left(\cos\left(HA\right)\right)}^{2}\sigma_{r}^{2} \end{array}$$

where the subcript *i* is skipped for the sake of readability. The Cartesian coordinates are mathematically correlated. The corresponding covariances read:

$$\begin{array}{l} \sigma_{XY}^{2}=\sigma_{YX}^{2}=\left(\cos\left(HA\right)\sin\left(HA\right)\sin^{2}\left(VA\right)\right)\sigma_{r}^{2}+\ldots \\ \left(r^{2}\cos\left(HA\right)\sin\left(HA\right)\cos^{2}\left(VA\right)\right)\sigma_{HA}^{2}-\left(r^{2}\sin\left(HA\right)\cos\left(HA\right)\sin^{2}\left(VA\right)\right)\sigma_{VA}^{2}\\ \sigma_{XZ}^{2}=\sigma_{ZX}^{2}=\left(-r^{2}\cos\left(HA\right)\sin\left(HA\right)\cos\left(VA\right)\right)\sigma_{HA}^{2}+\left(\cos\left(HA\right)\sin\left(VA\right)\cos\left(VA\right)A\right)\sigma_{r}^{2}\\ \sigma_{YZ}^{2}=\sigma_{ZY}^{2}=\left(-r^{2}\sin\left(HA\right)\cos\left(VA\right)\sin\left(VA\right)\right)\sigma_{HA}^{2}+\left(\sin\left(HA\right)\sin\left(VA\right)\cos\left(VA\right)\right)\sigma_{r}^{2} \end{array}$$

Consecutively, the transformed VCM $\hat{\Sigma }=F{\hat{\Sigma }}_{ori}F^{T}$ becomes fully populated.

#### 2.4.2. Simplification of the VCM

Because fully populated matrices may be uneasy to handle in LS adjustment, we propose, in this contribution, to approximate them following Zhao et al. [3]. The VCM will correspond to a gradual misspecification by neglecting mathematical correlations and heteroscedasticity:

(i) The approximated VCM ${\hat{\Sigma }}_{\left(i\right)}=F{\hat{\Sigma }}_{ori}F^{T}$ is used in the adjustment and further computation
(ii) The diagonal values of (i) are only considered and the approximated diagonal VCM ${\hat{\Sigma }}_{\left(ii\right)}$ is built as follows: $diag\left({\hat{\Sigma }}_{\left(ii\right)}\right)=diag\left({\hat{\Sigma }}_{\left(i\right)}\right)$.
(iii) The scaled identity matrix: ${\hat{\Sigma }}_{\left(iii\right)}=\sigma_{mean}^{2}I$ is used. The scaling factor $\sigma_{mean}^{2}$ is computed as the mean of the diagonal values of ${\hat{\Sigma }}_{\left(ii\right)}$. I is the identity matrix of size $\left(3n_{obs,}3n_{obs,}\right)$.

### 2.5. Test for Deformation

In the following, we assume that the same object is measured at two different epochs 1 and 2. Thus, two B-spline surface approximations are performed for two different point clouds. Based on information criterion results (Section 2.1), the optimal number of CPs used to approximate the point clouds may be different for each epoch.

#### 2.5.1. Test Statistic

As we aim to test for deformation, we define the null hypothesis $H_{0}$ and the alternative hypothesis $H_{1}$ as:

(10) $$H_{0}:E\left\{\Delta \right\}=0\;\mathrm{vs}.\;H_{1}:E\left\{\Delta \right\}\neq 0$$

$H_{0}$ states that no deformation occurs. We define $\hat{\Delta }$ as the difference between the two estimated surfaces at the two epochs given by $\hat{\Delta }=H\hat{\beta }$. H is a matrix which is splitted in two parts to provide a difference at the level of the gridded surface points. In the following, this test will be called test1.

In our particular case, the uniformly most powerful invariant test1 cannot be based on the estimated parameters. As aforementioned, these are the Cartesian coordinates of the CPs, which number may vary from epoch to epoch. Consecutively, we make use of the gridded surface difference and the corresponding test statistic reads:

(11) $$T_{apriori}=\hat{\Delta }\Sigma_{\hat{\Delta }\hat{\Delta }}^{-1}\hat{\Delta }=\frac{1}{\sigma_{0}^{2}}\hat{\Delta }Q_{\hat{\Delta }\hat{\Delta }}^{-1}\hat{\Delta }∼\chi_{u}^{2}\;\mathrm{with}\;\Sigma_{\hat{\Delta }\hat{\Delta }}^{-1}=H\Sigma_{\hat{\beta }\hat{\beta }}^{-1}H^{T}$$

where $\Sigma_{\hat{\beta }\hat{\beta }}^{-1}$ is the inverse of the VCM of the estimated CPs for both epochs and $\hat{\beta }=\left[\begin{array}{c} {\hat{p}}_{epoch1}\\ {\hat{p}}_{epoch2} \end{array}\right]$ contains the LS estimates of the CPs.

Using the apriori test statistic, the test decision is based on the quantile $k_{1-\alpha }^{\chi_{u}^{2}}$, i.e., on the $\chi_{u}^{2}$ test distribution with $u=n_{obs}-3\left(n+1\right)\left(m+1\right)$ degrees of freedom at the significance level $\alpha_{test1}$ Exemplarily, $H_{0}$ is accepted if $T_{apriori}\leq k_{1-\alpha }^{\chi_{u}^{2}}$.

The aposteriori test statistic is derived by replacing $\sigma_{0}^{2}$ by it’s aposteriori counterpart ${\hat{\sigma }}^{2}$ (Equation (4)) for each of the two approximations. From Teunissen [22], $T_{post}$ should theoretically follow a F-distribution. ${\hat{\sigma }}^{2}$ can be considered as a weighting factor of $T_{post}$ that accounts for functional and/or stochastic model misspecifications. Working with real data, it seems more appropriate to use this quantity rather than the apriori one.

For one set of observations, three values of the test statistics are obtained, which correspond to the three approximated stochastic models (i), (ii) and (iii). We call them $T_{post,(i)},T_{post,(ii)},T_{post,(iii)}$, respectively. By defining a significance level (α-level) as the probability of making the wrong decision when the null hypothesis is true and under the assumption that the F-distribution for the test statistic holds, we are able to reject or not the null hypothesis of test1.

Kermarrec et al. [4] shows for a real case study that accounting or not for mathematical correlations—up to neglecting them completely—affects strongly the test statistics of test1. The results were shown to depend on the scanning geometry (range and angle) and/or on the deformation magnitude. Test decision for small deformation close to the standard deviation of the range were more influenced by a misspecified stochastic model than when stronger deformation magnitude arised. A deeper analysis of the structure of the fully populated VCM by studying the ratio of the diagonal elements to the cross diagonal provided some mathematical explanations of these empirical conclusions.

#### 2.5.2. Bootstrap *p*-Values

Simplifications of the stochastic model will affect the previously defined test statistics. Indeed, from Equation (11), the test statistic $T_{post}$ depends on the VCM of the estimated parameters ${\hat{\Sigma }}_{\hat{p}\hat{p}}={\left(A^{T}{\hat{\Sigma }}^{-1}A\right)}^{-1}$ through $\Sigma_{\hat{\beta }\hat{\beta }}^{-1}$. As a consequence, neglecting mathematical correlations or misspecifying the heteroscedasticity of the raw observations may lead to an inappropriate rejection of the null hypothesis. To study more deeply the strength of this effect, we propose to test the significance of the deviation of the test statistics from a reference value. This reference value can be exemplarily computed with a more precise sensor, as proposed in Section 3. Indeed, the difference between the $T_{post,(i)},T_{post,(ii)},T_{post,(iii)}$ may not be significant enough to rigorously conclude that mathematical correlations could have been neglected without affecting the test decision. Thus, provided that a reference value for the test statistic $T_{post}$ is available, the significance of the difference can be easily assessed by computing the *p*-value of the so-called “0difference hypothesis test” called in the following test2: $H_{0}:E\left\{T_{post}-T_{ref}\right\}=0$

(12) $$\mathrm{vs}.\;H_{1}:E\left\{T_{post}-T_{ref}\right\}\neq 0$$

In the following, we call $T_{post}-T_{ref}=T_{0diff}$ the test statistic of test2. Thus, we have a total of three test statistics $T_{0diff,(i)},T_{0diff,(ii)},T_{0diff,(iii)}$ for the cases (i), (ii) and (iii) under consideration.

Provided that a distribution can be assessed to the test statistic of test2, the *p*-value can be computed from a reference table. However, if $T_{post}$ can be considered as F-distributed, the same cannot be said for $T_{ref}$, so that the distribution of $T_{0diff}$ is hardly tractable. Fortunately, bootstrap simulations provide an optimal and elegant way to estimate the critical values for such test statistics. In the following, we will shortly summarize the methodology to compute the bootstrap *p*-value according to McKinnon [23]. Without loss of generality, we introduce the procedure only for case (i). The computation for the two other cases is exactly similar.

We use a simple parametric bootstrap method in the sense of Efron [24], which can be summarized in four steps:

(1) Testing step: The first step starts with the approximation of scattered (TLS) observations from 2 epochs with B-spline surfaces. In the second step, a large number of observation vectors under $H_{0}$ have to be generated. We define a so-called bootstrap sample, as the mean of the surface differences, i.e., $S_{H0}=\frac{S_{2}-S_{1}}{2}$ considered as being generated under $H_{0}$ that no deformation occurs.
(2) Generating step: The generating step begins by adding to the generated bootstrap surface a noise vector, which structure corresponds to ${\hat{\Sigma }}_{\left(i\right)}$. We use a Cholesky decomposition of the VCM ${\hat{\Sigma }}_{\left(i\right)}=G^{T}G$ and generate a Gaussian random vector $W_{noise,i,k},i=1,2$ for the two epochs with mean 0 and variance 1 from the Matlab random number generator randn. The noise vector thus reads: $N_{i,k}=G^{T}W_{noise,i,k}$ Added to $S_{H0}$, we generate consecutively two noised surfaces, which we approximate with B-splines surfaces. Finally, we compute the aposteriori test statistics $T_{post}$. For one iteration $k_{BS}$, we call the corresponding test statistics $T_{post}^{k_{BS}}$.
(3) Evaluation step: $K_{BS}$ iterations are carried out. Following Davindson and McKinnon [25], we fixed $K_{BS}=999$. Finally, the *p*-value is estimated by $\hat{p}v_{HD}=\frac{1}{K_{BS}}\sum_{k_{BS}=1}^{K_{S}}I\left(T_{post}^{k_{BS}}-T_{ref}\right)$ according to McKinnon [23]. I is an indicator function, which takes the value 1 when $T_{post}^{k_{BS}}>T_{ref}$ and 0, on the contrary.
(4) Decision test: A large $\hat{p}v_{HD}$ indicates a large support of $H_{0}$ by the observations. Assuming that all assumptions were correct, $H_{0}$ is rejected if $\hat{p}v_{HD}<\alpha_{test2}$, where $\alpha_{test2}$ is the specified significance level, usually taken to 0.05.

**Figure 1:**
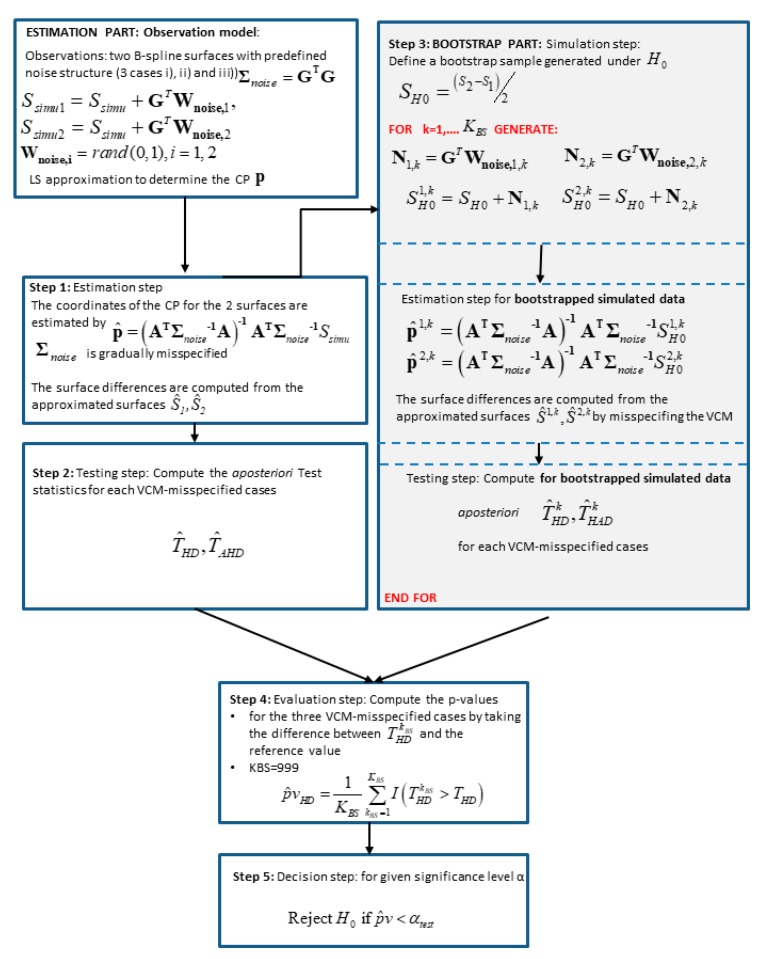
Flowchart describing the methodology to compute the *p*-value with a bootstrap approach.

The methodology of the bootstrapping approach is summarized in Figure 1.

### 2.6. Interpreting the p-Values

Whereas the alpha level of the test refers to a pre-chosen probability, the term *p*-value is used to indicate a probability that is calculated after the experiment was carried out and is often called an “observed significance level” for the test hypothesis (Wasserstein and Lazar [10]). Treating all assumptions to compute the *p*-value as being correct, the *p*-value can be interpreted as an empirical significance level, which can be compared to the chosen alpha level. More rigorously, the *p*-value is the probability that the chosen test statistic would have been at least as large as its observed value if every model assumption (e.g., correct data or study protocol) was correct, including the test hypothesis itself. Thus, a small *p*-value means rather that the data is more unusual if all the assumptions are correct, whereas a high *p*-value indicates that the data are not unusual under the statistical model. The *p*-value only measures the sample’s compatibility with the hypothesis. This slight difference with respect to a rough interpretation as “significance level” is worth mentioning to avoid misleading—and too strong—general conclusions about the impact of the stochastic model: an interpretation of the *p*-value as a “significance level” is only valid when all assumptions used to compute it can be considered as correct. In the context of this study, we will place ourselves within this framework, as we do not see where a wrong assumption may have been taken. However, since *p*-values remain only probability statements about the observed sample in the context of a hypothesis and not about the hypotheses being tested, we will avoid exaggerated general statements about the simplified stochastic model.

## 3. Case Study

In this section, we will apply the theoretical derivations of Section 2 for a real case study corresponding to a bridge under load observed with TLS and a highly precise point wise sensor, i.e., a laser tracker (LT). We test for the deformation magnitude of the gridded TLS point cloud between two epochs of load by approximating them with B-spline surfaces in order to carry out a rigorous test at the approximated surface level. Since different stochastic models will give rise to different values of the test statistics, we will investigate the significance of the difference with respect to a reference value by making use of the previously described bootstrap approach to compute accurate *p*-values. The results are expected to provide a support to judge if and when accounting for mathematical correlations can be replaced by a simplified stochastic model. However, as they are related to a specific case study and as mentioned in Section 2.4, general conclusions based on the interpretation of the *p*-values should be extended with a sense of measure and care.

### 3.1. Experiment Design and Data Acquisition

**Figure 2:**
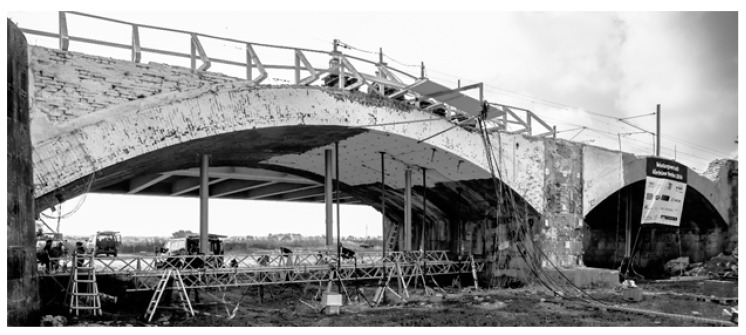
Side view from west of the arch 4 of the historic masonry arch bridge. The whitewashed area indicates the area of the direct influence of the load application. On the bridge: four hydraulic cylinders for the load application (Paffenholz et al. [27]). Reproduced with permission from Paffenholz, AVN; published by Wichmann-Verlag, 2018.

In this section, we use a real data set of an historic masonry arch bridge over the river Aller near Verden in Germany to compare and test for significance the results of the test statistics for detecting deformation at the level of the B-spline approximation. The historic masonry arch bridge was made of circular brick arches of following dimensions: width 14 m, depth 8 m and height 4–6 m. Figure 2 shows the side view from west of the arch 4 under investigation.

The aim of the experiment was the combination of numerical models and experimental investigations for model calibration (Schacht et al. [26]). An interdisciplinary project team with partners from industry and academia has carried out two load tests with a maximum load of 570 ton in March and June 2016. The contributions from researchers of the Geodetic community were the detection of load-induced arch displacements by means of, e.g., laser scanner, laser tracker and ground based synthetic aperture radar, which is discussed, e.g., in Paffenholz et al. [27]. The 3D point cloud acquisition was carried out using TLS sensors of kind Zoller+Fröhlich (Z+F) Imager 5006/h in periods of a constant load on the bridge. 3D point clouds for different load scenarios ranging from 1 up to 6 meganewton were captured and finally processed.

In the scope of the load testing the standard load of 1.0 meganewton should be clearly excited. By this setup first nonlinear deformations should be detected. According to Schacht et al. [26] and the references therein, performed numerical simulations stated that a loading with five-times the standard load had to be realised. Thus, a maximum load of approximately 6.0 meganewton was defined, produced by four hydraulic cylinders. These hydraulic cylinders were mounted on the arch (see Figure 2). Injection piles of length 18 m in depth realized the counteracting force and threaded rods, the connection of hydraulic cylinders and injection piles. A detailed description of the bridge structure as well as the design of experiments can be found in (Schacht et al. [26]).

The acquisition of the 3D point clouds was carried out from a fixed laser scanner position for the various load steps. To support the subsequent calculation and interpretation of deformation measurements, a 3D point cloud filtering with respect to objects on the arch surface was performed. Consecutively, interfering objects (other sensor installations like prisms for the laser tracker and strain gauges), which most likely appear differently in various load steps were carefully removed from the 3D point clouds. Previous investigations have shown, that aforementioned objects appear differently in the load steps and could lead to misinterpretations in the subtraction of a load step with respect to a reference epoch, see (Table 2 in Paffenholz et al. [27]).

The measurements of the laser tracker to pre-installed prism well distributed in the arch of the bridge serve as reference measurements. In particular, the measurements to the prisms denoted by L8, L10 and L13 are used as the reference for the computation of the *p*-values as proposed in Section 2.

In this contribution, we will consider the five epochs of loads for further analysis: the reference (no load) is called E00 whereas the fifth one (E05) corresponds to the maximum load, i.e., maximum deformation. Thus, we have a total of five deformation steps corresponding to five5 load steps, which we call Def1 between E00–E01, Def2 between E00–E02, Def3 between E00–E03, Def4 between E00–E04 and Def5 between E00–E05. As the load step, one deformation step is defined as the deformation difference between one epoch of load and the reference one.

Because the weights were applied in the middle of the bridge, specific parts were shown to have been more strongly and rapidly influenced by the loading than others. Consecutively, we expect the impact of mathematical correlations to be lower for parts located close to the extremity of the bridge. This is due both to the smaller deformation magnitude and the scanning geometry, following the interpretation of Kermarrec et al. [4].

#### 3.1.1. Surface Approximation

**Figure 3:**
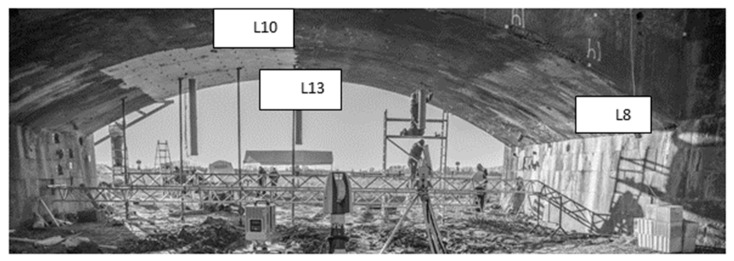
Representation of the bridge under load with the localization of the three patches L13, L10 and L8. The load was positioned approximately in the middle of the bridge under which the TLS was positioned (image adapted from Paffenholz et al. [27]). Reproduced with permission from Paffenholz, AVN; published by Wichmann-Verlag, 2018.

**Figure 4:**
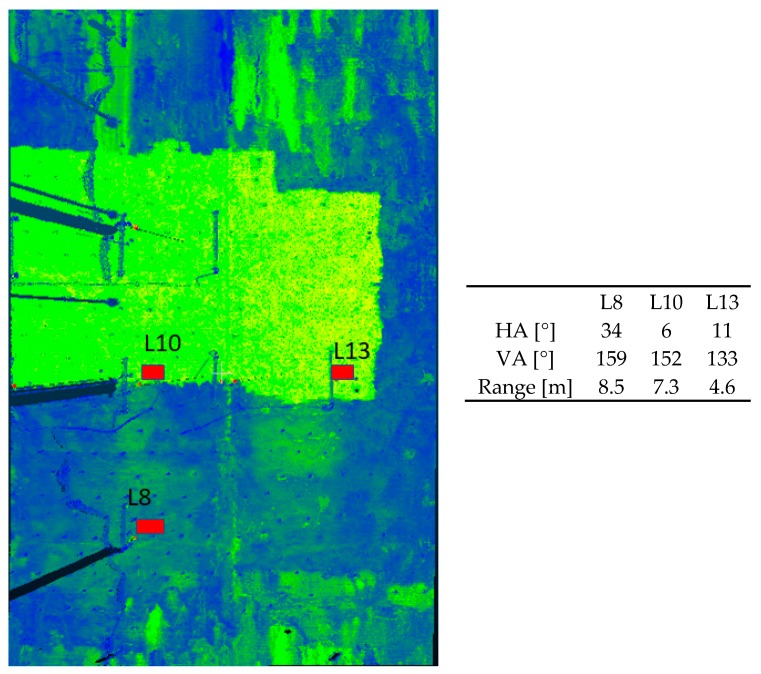
Left: Localisation of the TLS surfaces (red rectangles) and corresponding LT points. Right: mean HA, VA in [°] and range for the surface under consideration.

As proposed in Section 2, deformation magnitudes from B-spline approximation are assessed by taking the differences between the gridded surfaces obtained at two different epochs of load. In order to have an optimal functional model, we did not make use of a global approximation of the point cloud from the whole bridge but selected small patches around the reference LT points. Using the software CloudCompare, the same surfaces in each point cloud for the reference epoch and the 5 epochs of load under consideration were selected. They were located in the direct neighbourhood of the three prisms observed by the LT. Figure 3 shows the localisation of the chosen surfaces around the points L8, L10 and L13. These points were intentionally chosen. Indeed, for L8 and L13, the deformation magnitudes of approximately 4 mm are small and comparable but the surfaces are recorded under two different scanning geometries. Small deformation magnitudes of the order of the standard deviation of the noise are highly interesting since they may not be detected with the statistical tests. L10 serves as a reference point: it has a stronger deformation magnitude of 10 mm and is scanned under a favourable geometry (see also Figure 4, right).

The corresponding extracted point clouds were gridded in 10 cells in both directions, leading to a total of 100 gridded observations for the B-spline approximation. All observations falling in one cell were correspondingly averaged. We chose intentionally a loose gridding so that only a small amount of observations is available. This allows a better comparison between the deformation magnitudes obtained with the B-spline modelization and the one obtained from the point wise LT measurements. Indeed, increasing the number of points available by reducing the gridding leads to the modelling of small artefacts of the surface that could affect the computed surface difference when compared with a value from a point-wise LT. The comparison between sensors and methods (point wise versus surface wise) is, thus, made more trustworthy. On the contrary, higher gridding, would not affect the present results as soon as enough points are available to perform a B-spline approximation with LS. The values for the test statistics would change accordingly, the distance between two point clouds being impacted by the number of points.

In order to mathematically approximate the small patches with B-spline surfaces, a parameterization was carried out using a uniform method, which is justified by their relatively smooth and uncomplicated geometries. The knot vector was determined as proposed in Section 2.1. The number of CP was determined using the BIC criterion (Zhao et al. [3]). Whereas for L8 4 CP in both directions were found as optimal, 4 in the u-direction and 3 in the v-direction were considered for L10. The same values were found for the bootstrap mean surfaces and for all epochs under consideration.

Our goal being to test the impact of mathematical correlations for B-spline surface approximations, we do not use a Gauss-Helmert Model (Lenzmann and Lenzmann [28]) to approximate a planar surface, which may not correspond exactly to the underlying geometry of the point cloud.

#### 3.1.2. Stochastic Model for TLS

Figure 4 (left) shows the localization of the three LT points chosen in this contribution. The TLS was positioned approximately in the middle of the bridge. Consecutively, whereas L10 can be considered as optimally scanned at a short distance in the Up-direction, L8 was scanned with a less favourable geometry with respect to footprint, range and incidence angle. The stochastic model for the B-spline approximation will, thus, differ for both points. Figure 4 (right, table) gives the corresponding HA, VA and ranges for the points under consideration (coordinates in the local TLS coordinate system).

Following the proposal of Section 2.2, we use the intensity model to compute the standard deviation of the range. Whereas a large mean intensity of 1,557,500 Inc for the zero-load epoch (E00) was found for L10, leading to $\sigma_{\rho }^{L10}\approx 0.47\;\mathrm{mm}$, the mean of the intensity for L8 reaches 99,874 Inc so that $\sigma_{\rho }^{L8}\approx 2.20\;\mathrm{mm}$. For L13, a value of $\sigma_{\rho }^{L13}\approx 0.48\;\mathrm{mm}$ corresponding to a mean intensity of 1,468,652 Inc was computed. All range variances were computed using Equation (8) with two significant digits to show the slight difference between L13 and L10 due to the range (Figure 4, right, table). The range variances were evaluated similarly for the five load epochs under consideration. A maximum difference of ±0.10 was found compared with the E00 values.

Temporal correlations between points are considered as meaningless due to the gridding approach, which do not allow for the determination of a time stamp for averaged observations. Thus, we only consider the heteroscedasticity of the raw TLS observations. Accounting for temporal range correlations acting as decreasing the variance (Kermarrec et al. [29]), we will investigate the impact of lower values on the *p*-values for the sake of completeness. In a first approach, the angle variances are chosen to correspond to the manufacturer datasheet.

We adopt the strategy developed in Section 2. Thus, three cases are considered corresponding to a gradual misspecification of the approximated VCM. We compute the corresponding test statistics for deformation as well as the *p*-values.

#### 3.1.3. Stochastic Model for LT

In order to test the difference between the obtained test statistics for significance, a reference value is needed. We propose to use the deformation magnitude obtained with the highly precise LT, thus considering a point-wise distance as a reference value with respect to the deformation magnitudes obtained from the TLS point cloud approximations with B-spline surfaces.

We define $T_{ref}$ as:

(13) $$T_{ref}={\left(L_{i,E00}-L_{i,E0j}\right)}^{T}{\hat{\Sigma }}_{L,j}^{-1}\left(L_{i,E00}-L_{i,E0j}\right),i=8,10,13,j=1,\ldots ,5$$

with $L_{i,E00}-L_{i,E0j}$ being the Cartesian coordinate difference between the LT point *i* at epoch E00 and the same LT point at epoch E01, E02, E03, E04 and E05, respectively.

Since repeated LT measurements were carried out for all epochs under consideration, we make use of the aposteriori variance factors (Equation (4)) of the performed adjustment to estimate the LT coordinates (i.e., a mean with $n_{obs\_LT}=3$ repetitions). We follow, thus, the definition of $T_{post}$. ${\hat{\Sigma }}_{L,j}$ corresponds to the aposteriori estimated VCM of the coordinate difference $\left(L_{i,E00}-L_{i,E0j}\right)$ with j=1,…,5. ${\hat{\Sigma }}_{L,j}$ reads for the LT point i:

(14) $${\hat{\Sigma }}_{L,j,i}=\sigma_{0,LTi}^{2}\left[\begin{array}{ccc} {\hat{\sigma }}_{x,E00}^{2}+{\hat{\sigma }}_{x,E0j}^{2} & 0 & 0\\ 0 & {\hat{\sigma }}_{y,E00}^{2}+{\hat{\sigma }}_{y,E0j}^{2} & 0\\ 0 & 0 & {\hat{\sigma }}_{z,E00}^{2}+{\hat{\sigma }}_{z,E0j}^{2} \end{array}\right]$$

with ${\hat{\sigma }}_{x,E00}^{2}$ and ${\hat{\sigma }}_{x,E0j}^{2}$ being the mean values of the variances of the x-component from the repeated measurements for epoch E00 and E0j, respectively, divided by $n_{obs\_LT}-1$. The other variances are defined similarly for the *y*- and *z*-components. As these values were computed without any apriori weighting, we multiply them with $\sigma_{0,LTi}^{2}$, i.e., the apriori variance for the LT point *i* under consideration, which we take according to manufacturer specification (15 µm + 0.6 µm/m). Please note that the subscript *i* is switched in Equation (14) for the sake of readability.

**Figure 5:**
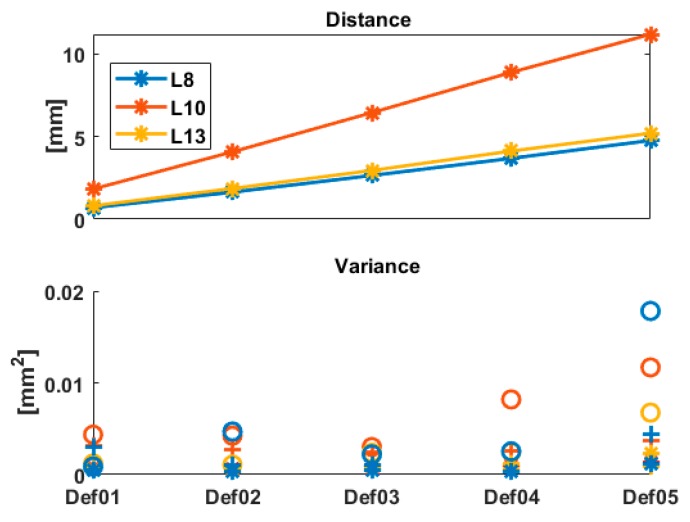
Top: Euclidian distance in [mm] between the epochs under consideration for LT measurements (Def1, Def2, Def3, Def4, Def5). 3 LT points are considered: L8, L10 and L13. Bottom: corresponding apriori variance of the distance difference in [mm2]: * for *x*-, + for *y*- and o for *z*-component, respectively.

The Euclidian distance between the points, as well as the variance of the *x*-, *y*- and *z*-components, is summarized in Figure 5 for the 3 points under consideration. As can be seen, the variance of the coordinates is generally increasing with increasing distance. The differences found between Def02 and Def03 are below 0.025 mm^2^ (i.e., at the submm level for the corresponding standard deviation) cannot be considered as significant enough to draw conclusions from them. The variance for the z-component is higher than for the other components, as it is strongly depending on the range variance of the raw measurements, which is higher than the angle variances (see Equation (9)). The points L13 and L8 have the same deformation magnitude under load. However, as these 2 points correspond to two different geometries (Figure 4, right), the corresponding observations have, thus, different variances for the *x*-, *y*- and *z*-components. Similarly, the intensity values of the TLS measurements led to different standard deviations of the range. These two results are coherent.

For the sake of shortness, the values of $T_{ref}$ are not presented here. Similarly to the behavior highlighted in Figure 5, they increase with the deformation magnitude and are always over the critical value of the F-distribution with α=0.05. Consecutively, the highly precise LT allowed for the acceptance of the $H_{0}$ hypothesis with the chosen confidence level, i.e., the detection of deformation for all three points under consideration. This result holds true even for L8 and L13 and the Def01 step for which the magnitude was around 0.5 mm.

### 3.2. Results

**Figure 6:**
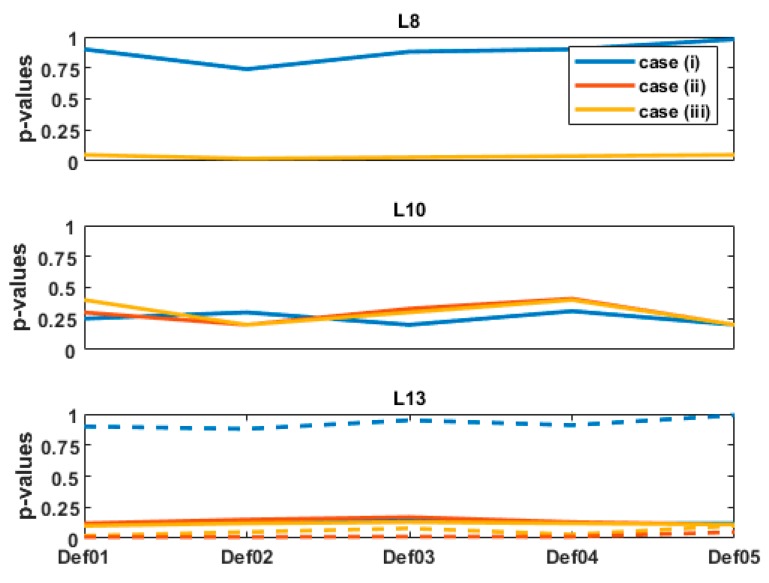
*p*-Values obtained for L8 (top), L10 (middle) and L13 (bottom) for the three stochastic model ((i), blue accounting for mathematical correlation), (ii), diagonal values of (i) and (iii), scaled identity matrix). The values obtained for the 5 deformation cases Def01, 02, 03, 04, 05 corresponding to increasing load are linked by a line for the sake of readability. The dotted line for L13 (bottom) corresponds to the values that would have been obtained by artificially increasing $\sigma_{\rho }$ to 5 mm.

Figure 6 presents the results obtained for the *p*-values for the 5 deformation steps under consideration. For L8 (Figure 6 top), a high *p*-value—larger than 0.9—is obtained for case (i), for which mathematical correlations and heteroscedasticity were taken into consideration. This is a strong evidence not to reject H0, and means that the difference between $T_{ref}$ and $T_{post,i}$ cannot be considered as significant, provided that all assumptions for test2 are correct (non-corrupted observations, correct model and adequate study protocol).

On the contrary, 9 times smaller *p*-values are found for both cases (ii) and (iii). For the computed aposteriori $T_{ref}$, the *p*-values were around and slightly lower than $\alpha_{test2}=0.05$. Consecutively, it cannot be concluded, as for case (i), that the difference between $T_{ref}$ and $T_{post}$ are non-significant. Clearly, different values of $T_{ref}$ may have led to different *p*-values. However, within a range of plausible values, these would not have changed the high deviation to $T_{post,i}$.

For L13 and L10 and although the deformation magnitudes differ strongly (Figure 5, top), the *p*-values are similar for the 3 stochastic models under consideration, independently of the deformation step. For the $T_{ref}$, the *p*-values are, in all cases and for both LT points, higher than $\alpha_{test2}=0.05$ by a factor 5 for L10 and 2.2 for L13. Thus, the difference between $T_{ref}$ and $T_{post}$ cannot be considered as significant. Additionally, the *p*-values that would have been obtained by taking 10 times the original standard deviation of the TLS range are plotted as dotted line in Figure 6 (bottom). It aims to highlight the strong impact of $\sigma_{\rho }$ on the *p*-values, which we further discuss in the next section.

**Figure 7:**
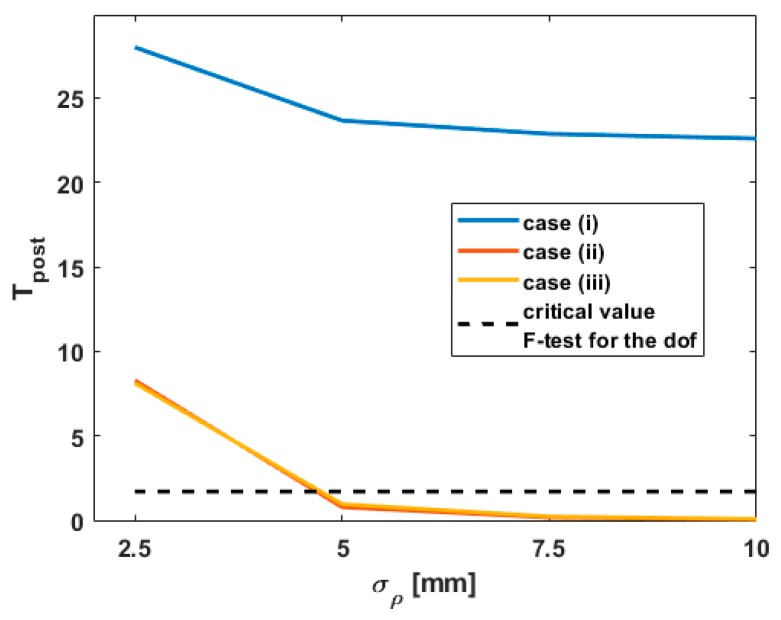
$T_{post}$ obtained for L8 by varying $\sigma_{\rho }$ from 2.5 to 10 mm.

Since the values of $T_{post}$ were over the critical values of the F-distribution for $\alpha_{test1}=0.05$ in all cases, they are not shown for the sake of shortness. They are replaced by the behavior of $T_{post}$ by misspecifying $\sigma_{\rho }$ for L8, starting from the value of 2.5 mm computed with the intensity model. The corresponding results are presented in Figure 7. Similar conclusions were obtained for the other points L10 and L13.

Please note that the same behavior is obtained by replacing $T_{post}$ with $T_{prio}$ due to the optimal functional model obtained for the small patches under consideration.

### 3.3. Discussion

#### 3.3.1. Role of $\sigma_{\rho }$ on the *p*-Values

The combination of Figure 5 and Figure 6 highlights that mathematical correlations should not be neglected, particularly as $\sigma_{\rho }$ increases. Indeed, the cases of Figure 5 (top, L8) and Figure 5 (bottom, L13) correspond to similar deformation magnitudes (see Figure 4, L8 and L13). However, the *p*-values strongly differ for both cases, which can be linked with the different values of $\sigma_{\rho }$. Whereas the *p*-values are high for L8 and far over $\alpha_{test2}=0.05$, they are much lower for L13. For the computed $T_{ref}$, they are found around and lower than $\alpha_{test2}=0.05$, i.e., the difference with $T_{ref}$ can be considered as significant for case (ii) and (iii), but not for case (i). As this latter corresponds to a more optimal stochastic model (temporal correlations were neglected due to the gridding), it highlights the impact of the chosen simplification.

Moreover, the *p*-values for L13 exhibit similar values in all three cases (i), (ii) and (iii) and, are, thus, independent of the underlying stochastic model. This is not the case for L13 if $\sigma_{\rho }$ is misspecified (i.e., higher than assumed) as shown in Figure 6 (bottom, dotted line). This result confirms the role of increasing $\sigma_{\rho }$ on the *p*-values. For an optimal $\sigma_{\rho }$ computed with the intensity model, mathematical correlations can be consecutively neglected without affecting the significance of the difference between $T_{ref}$ and $T_{post}$. This is an important result, that should, however, not be too rapidly generalized. Indeed, not only the range variance but also the geometry of the scanning plays an important role. Kermarrec et al. [4] show that variations of HA or VA can lead to high values of the cross-diagonal with respect to the diagonal values of the fully populated VCM (see Equation (9) and following). However, if the point cloud is recorded from a TLS under optimal geometric conditions with a $\sigma_{\rho }$ under 2.5 mm (Figure 7), we propose to neglect mathematical correlations by making use of the simplification corresponding to case (iii). The corresponding VCM is diagonal with a constant variance factor that can be easily computed from the transformed VCM. The LS computation is simplified and more stable as no inverse of the fully populated VCM has to be performed. This is of great advantage for large point clouds.

#### 3.3.2. Role of $\sigma_{\rho }$ on $T_{post}$

As aforementioned, the range variance is playing a central role in the computation of $T_{post}$ (Figure 7). As $\sigma_{\rho }$ decreases, $T_{post}$ falls under the critical value of the F-distribution for the given degree of freedom (75 and 37) with $\alpha_{test1}=0.05$. Thus, the $H_{0}$ of test1 is accepted. Neglecting mathematical correlations (case (ii) and (iii)) by misspecifying at the same time the range variance can lead to the acceptance of the $H_{0}$ hypothesis “no deformation”, although highly precise sensors such as LT were able to detect a deformation with a high confidence. On the contrary, accounting for mathematical correlations allows the rejection of $H_{0}$, which is a more trustworthy test decision. This behavior strongly supports that accounting for correlations—mathematical or temporal—has a similar effect as decreasing the range variance in a diagonal model (Kermarrec and Schön [5]). Thus, it is possible to find an optimal range variance for which the results are comparable with the one obtained with a fully populated VCM. The so-called diagonal correlation model (DCM) proposed in Kermarrec et al. [29] for temporal correlations could be used to simplify the computational burden associated with a fully populated VCM. Its generalization for mathematical correlations needs, however, further investigations.

## 4. Conclusions

The modelization of TLS point cloud with B-spline surfaces provides a rigorous framework to test for deformation. Indeed, recently available software allows for the visualization of deformation by means of maps of distances, but not for statistical testing. However, the usual congruency test cannot be used with B-spline surfaces since the number of parameters to estimate at the two epochs may differ strongly. Consecutively, a specific test statistic had to be developed, which is based on the difference of the gridded approximated B-spline surfaces. The test distribution can be found as corresponding to the F-distribution for the aposteriori test statistic.

In order to get a trustworthy test decision, i.e., the rejection of the null-hypothesis that no deformation occurs, an optimal stochastic model for the surface difference is necessary. Provided that temporal correlations are neglected, the model relies mainly on the heteroscedasticity of the raw TLS observations. Assuming that the angles have a constant variance provided from the manufacturer, the range variance can be estimated with an empirical intensity model that allows to account for specific effects such as scanning geometry or atmospheric condition in an optimal way. Since Cartesian coordinates are needed for the B-spline surface approximation, mathematical correlations have to be considered, leading to a fully populated VCM of the transformed raw measurements. In this contribution, the impact of neglecting mathematical correlations on the results of the developed test statistics for deformation was investigated. Moreover, by means of a bootstrap approach, it was tested if the test statistics difference obtained by varying the stochastic model with respect to a reference value obtained from a LT could be considered as significant or not. To that aim, *p*-values were computed for optimal or less optimal stochastic models. Using real data from a bridge under load, three LT points were chosen. In the neighbourhood of these LT points, rectangular patches of observations were extracted for 5 load steps and optimally approximated with B-spline surfaces. The test statistics and *p*-values were computed for three stochastic models: (i) with mathematical correlations and heteroscedasticity, (ii) the diagonal version of (ii) and (iii) a scaled identity matrix.

The strong dependency of the *p*-values with the chosen standard deviation of the range was highlighted by using two LT points corresponding to a similar deformation magnitude under load but different scanning geometries. It could be shown that the significance of the difference of the test statistics and the LT reference value was decreasing with the range variance. A limit value around a range standard deviation of 2.5 mm could be identified. Over this value, mathematical correlations should not be neglected for a trustworthy test decision. The results of this work on test statistics can be used as soon as mathematical correlations due to the transformation from polar raw to Cartesian measurements have to be considered in a LS adjustment. In order to simplify the computational power linked with the fully populated VCM, a proposal was made to decrease artificially the range variance in order to get an easy to handle diagonal VCM. This strategy needs, however, further investigations to be widely extended, which we let to a next contribution.
